# Employees Receiving Inpatient Treatment for Common Mental Disorders in Germany: Factors Associated with Time to First and Full Return to Work

**DOI:** 10.1007/s10926-021-09985-4

**Published:** 2021-05-29

**Authors:** Alexandra Sikora, Gundolf Schneider, Uta Wegewitz, Ute Bültmann

**Affiliations:** 1 Division 3 Work and Health, Federal Institute for Occupational Safety and Health (BAuA), Nöldnerstr. 40-42, 10317 Berlin, Germany; 2 Department of Health Sciences, Community and Occupational Medicine, University of Groningen, University Medical Center Groningen, Groningen, The Netherlands; 3 Division Rehabilitation Research, Federal German Pension Insurance, Berlin, Germany

**Keywords:** Return to work, Mental disorders, Psychiatric hospitals, Rehabilitation centers, Survival analysis

## Abstract

*Purpose* In Germany, return to work (RTW) after inpatient treatment for common mental disorders (CMDs) is a complex process at the intersection of the mental healthcare system and the workplace. This study examined (1) the time to first and full RTW and (2) associated factors among employees receiving inpatient treatment for CMDs. *Methods* In this prospective cohort study, employees receiving inpatient psychiatric or medical rehabilitation treatment for CMDs were interviewed by phone during their last week before discharge. Follow-up interviews were conducted after 6, 12, and 18 months. Health-, personal, and work-related factors were used from baseline measurement. Parametric survival analysis was conducted to identify factors associated with time to first and full RTW. *Results* A total of *N* = 269 participants who stayed at a psychiatric clinic or a medical rehabilitation facility were included. Almost all participants (*n* = 252, 94%) from both treatment settings reported a first RTW and a full RTW. The time to first and full RTW was shortest among participants from medical rehabilitation (both median 6 days) and longer among participants from psychiatric treatment (median 17 days to first RTW and 73 days to full RTW). While only health-related and personal factors were associated with time to first RTW, leadership quality and needed individual RTW support were associated with time to full RTW. *Conclusions* More attention to work accommodation needs for RTW in clinical practice and coordinated actions towards RTW in collaboration with key RTW stakeholders in the workplace may support a timely RTW.

*Clinical Registration Number* DRKS00010903, retrospectively registered.

**Supplementary Information:**

The online version contains supplementary material available at 10.1007/s10926-021-09985-4.

## Introduction

Mental health problems are a matter of great concern among the working-age population, with enormous burdens and costs for the affected individuals, their families, colleagues, employers, and society at large [1–4]. In Germany, 112 million sickness absence days and more than 71,000 new disability pensions were related to mental disorders in 2018 [5, 6]. Mood (F30–F39), neurotic, stress-related, and somatoform disorders (F40–F48) had, with nearly 90%, the highest share in sickness absence days among all mental disorders [7]. The longest sickness absence durations among all mental disorders were due to depressive disorders (93 days per 100 insured persons) and adjustment disorders (51 days per 100 insured persons) [7].

For patients with common mental disorders (CMDs), two main inpatient treatment settings are available in Germany: psychiatric clinics and medical rehabilitation facilities. These health service providers differ primarily regarding their funding (psychiatric clinics by statutory health insurances versus medical rehabilitation by the German Pension Insurance) and their primary goals. Whereas inpatient psychiatric clinics help mainly in acute crises, medical rehabilitation facilities aim to improve work and daily life capacity [8]. Nevertheless, the pathways into these treatment settings vary and are often decided upon personal needs and medical or social insurance recommendations [9].

To support recovery and prevent negative consequences like job loss or disability retirement [10, 11], return to work (RTW) is a complex but essential process at the intersection of the mental healthcare system and the workplace [12]. RTW provides financial security, a structured daily routine, promotes social interaction, and strengthens self-efficacy [2, 13]. According to Young et al. [14], RTW is a dynamic process consisting of four phases: (1) off work, (2) re-entry, (3) maintenance and (4) advancement. Based on this approach, phase-specific outcomes during the entire RTW process can be defined and evaluated, such as time to first RTW and time to full RTW.

Previous research on prognostic factors relating to the time to RTW after CMDs focused primarily on personal (e.g., RTW expectations or RTW self-efficacy) and health-related (e.g., sickness absence duration or symptom severity) factors [15–17]. While several work-related risk factors have been associated with sickness absence and work disability (e.g., high physical/psychosocial job demands, low levels of work organisation and support, negative beliefs, and workplace attitudes) [18–20], the role of work-related factors for RTW has been insufficiently examined [21, 22]. In particular, evidence of work-related factors, such as social support from colleagues and supervisors, job demands, and work accommodations for RTW (e.g., temporary workload reduction or individual RTW support) is inconclusive or still lacking [17, 22–24]. Like communication between employee and employer or social support, some work-related factors can act as facilitators and barriers for RTW [25]. A qualitative meta-synthesis showed that employees with CMDs favour a gradual RTW and that low social support from supervisors and colleagues during RTW complicates the realisation of work accommodations [26]. Moreover, as most previous research was conducted in other jurisdictional contexts, particularly Scandinavia and the Netherlands, evidence from Germany, which has its own social security system [27], is needed. Therefore, the present study aims to examine (1) the time to first RTW and full RTW among employees after inpatient treatment for CMDs in Germany and (2) the health-, personal, and work-related factors that are associated with time to first and full RTW, with a specific focus on work accommodation needs for RTW.

## Methods

### The German Mental Healthcare System

Germany has a comprehensive statutory social security system that is collectively financed by employers' and employees' compulsory contributions. Within the social insurance sector, statutory health insurances and the German Pension Insurance are two important funding agencies [28]. This pluralism of institutions can be found at various levels (e.g., service providers, professions, settings, or funding) in the German mental healthcare system and adds to its fragmentation [28–30]. Although mental healthcare services are accessible without significant financial barriers, utilisation rates are relatively low [31]. As Bode [28] states ‘*Overall, German citizens have various opportunities to receive rehabilitation services, but must often grapple with a jungle of institutions before accessing them’* (p. 52)*.* In addition to the pluralism of institutions and services, pathways into the treatment settings often remain unclear and do not follow explicit selection guidelines [9].

Two essential pillars of mental healthcare in Germany are inpatient psychiatric treatment and medical (psychosomatic) rehabilitation [32, 33]. Although the primary care goals differ, the diagnostic spectrum does not clearly distinguish between both settings [33], particularly with regard to CMDs. Due to the strong specialisation by indications and the specialist principle, medical rehabilitation in Germany is more strongly oriented towards acute care than it is in other European countries [34]. In contrast to most European countries, medical rehabilitation in Germany is dominantly carried out as a 3- to a 5-week inpatient treatment programme in specific facilities away from home, and access has to be claimed by the patient and granted by the German Pension Insurance [34]. Admission for psychiatric treatment can only take place through a physician [33]. More detailed information about the jurisdictional context concerning sickness absence benefits in Germany is provided elsewhere [27].

### Study Design and Setting

This prospective cohort study was part of a larger mixed methods follow-up study with a qualitative sub-sample on ‘returning to work after sickness absence due to common mental disorders’ at the Federal Institute for Occupational Safety and Health [27, 35]. The present study focuses on the quantitative study sample and addresses the quantitative research aims.

In terms of the outcome of interest and feasibility, it was decided to only recruit participants from an inpatient treatment setting [27]. Because of the lived practice in Germany, where both acute psychiatric clinics and medical rehabilitation facilities belong to the main mental health services, and both offer inpatient treatment services, they were chosen as the entrée for study participation.

Through five cooperating clinics (two psychiatric clinics and three medical psychosomatic rehabilitation facilities), sick-listed employees receiving inpatient psychiatric or medical rehabilitation treatment for CMDs were recruited between August 2016 and November 2017. For baseline measurement, participants were interviewed by phone during the last week before clinical discharge. Follow-up phone interviews were conducted after 6, 12, and 18 months. Inclusion criteria were: age between 18 and 60 years, previous sickness absence duration no longer than six months within the last 12 months, part- or full-time employment, permanent or fixed-term employment for at least 18 months, intention to RTW with the present employer, and treated for a first medical diagnosis and maximally one further diagnosis of the following disorders: depressive disorder (F32.0, F32.1, F32.2), recurrent depressive disorder (F33.0, F33.1, F33.2), agoraphobia with panic disorder (F40.01), panic disorder (F41.0), generalised anxiety disorder (F41.1), mixed anxiety and depressive disorder (F41.2), or an adjustment disorder (F43.2). Ethical approval for the study was granted by the Hannover Medical School (MHH) Ethics Committee (ID: 3211–2016). All participants provided written consent.

### Outcomes

**Fig. 1:**
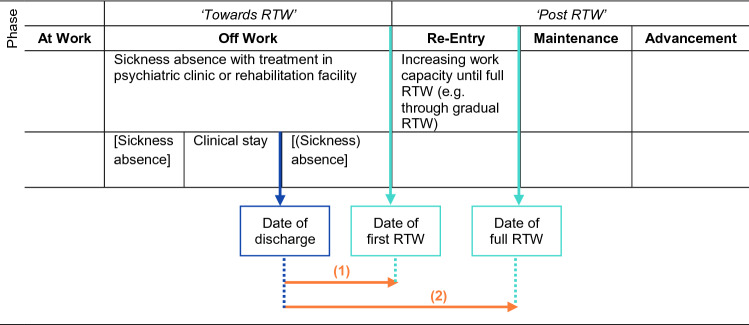
Overview of the RTW phases and outcomes based on Young et al. [14]

The outcome measures were time to first RTW and time to full RTW in calendar days, as reported by the participants (Fig. 1).

1. Time to *first RTW* was defined as the duration starting from the date of clinical discharge until the first day back at work.
2. Time to *full RTW* was defined as the duration starting from the date of clinical discharge until the point in time when the re-entry phase was completed with a return to the same number of working hours as before the sickness absence (or just reduced in general by employment contract) and maintaining this status for at least 28 days [36].

### Health-, Personal and Work-Related Factors

Except for the medical diagnoses that were reported by clinicians, the factors were self-reported at baseline measurement. Translated and/or validated questionnaires were used whenever possible. A detailed overview of all used questionnaires and measurement points is provided elsewhere [27].

#### Health-Related Factors

Previous sickness absence and treatment duration were reported in weeks. Additionally, the sum of previous sickness absence and treatment duration as ‘length of absence period from work’ was computed. Self-rated health was assessed with the question ‘How would you describe your current health?’ [37] and was dichotomised into ‘*poor*’ (*poor, bad*) versus ‘*good*’ (*satisfactory, good, very good*) [38]. Depressive symptoms were measured with the 8-item Patient Health Questionnaire [39]; scores range from 0 to 24, with higher scores indicating more depressive symptoms. The first medical diagnosis given by clinicians according to the ICD-10 code system was categorised into two groups: ‘*depression or anxiety disorder*’ (F32.0–F33.2, F40.01, F41.0–F41.2) and ‘*adjustment disorder*’ (F43.2). Medical treatment after clinical discharge (*no/yes*) was measured. Work-relatedness of disorder was assessed on a 5-point Likert scale from (1) ‘*not at all*’ to (5) ‘*extremely*’ [40].

The ‘work orientation and RTW preparation during treatment’ was assessed with four descriptive questions (*no/yes*) adapted from Wienert and Bethge [41].

#### Personal Factors

RTW expectation was assessed with a single question (‘When do you expect to return to your previous workplace considering your current state of health—within the next 3, 6, 9, or 12 months, or no return to the previous workplace?’) adapted from SIBAR [42]. The answer was assessed in 3-month categories and dichotomised into ‘expected RTW ≤ 3 months’ (*positive RTW expectation*) versus ‘expected RTW > 3 months or no return to previous workplace’ (*negative RTW expectation*) [43]. RTW self-efficacy (RTW-SE) was measured with a translated version of the RTW-SE questionnaire by Lagerveld et al. [27, 44]. Scores range from 1 to 6, with higher scores indicating higher perceived self-efficacy regarding RTW. Additionally, the RTW-SE scale was dichotomised into ‘*low RTW-SE* ≤ *4.5*’ and ‘*high RTW-SE* > *4.5*’ based on a very similar median split [45]. Work ability was assessed as a single item score from 0 ‘*completely unable to work*’ to 10 ‘*work ability at its best*’ [46].

#### Work-Related Factors

Company size (*small/large* ≥ *250 employees*), sector (*private/public*), working in shifts (*no/yes*) and being a civil servant (*no/yes*) were included as job characteristics. Quantitative demands, work-privacy conflict, influence at work, degrees of freedom at work, meaning of work, quality of leadership, social support from colleagues and supervisor, sense of community and mobbing (single item) were measured with the Copenhagen Psychosocial Questionnaire (COPSOQ) [47, 48]. All questions related to the last job before the clinical stay. COPSOQ scores range from 0 to 100 and for mobbing from 1 to 5.

#### Work Accommodation Needs

From the ‘ten most important necessary work accommodation needs for RTW’ [27], which were measured as self-formulated items, the following work accommodation needs were included: gradual RTW, workload reduction, individual RTW support, and change of workplace (all *no/yes*).

#### Covariates

Age, sex, and socioeconomic status were included. Socioeconomic status was aggregated as a multidimensional index of education, occupation, and household income; scores range from 3 to 21, with higher scores indicating higher socioeconomic status [49].

### Statistical Analysis

The statistical analyses were performed for *N* = 269 participants who completed at least one follow-up measurement. From the original baseline sample [27], *n* = 17 participants (*n* = 12 from psychiatric setting; *n* = 5 from rehabilitation setting) dropped out directly after baseline measurement and were therefore not included in the present study. These 17 participants were on average younger, lived more often without a partner, smoked more frequently, reported a higher work-privacy conflict and higher depressive symptoms. The overall response rate of participants who completed all follow-up measurements was 91% (*n* = 259). A detailed flowchart of study participation is provided elsewhere [27].

First, descriptive statistics were calculated for the proportions of participants who achieved a first RTW and full RTW within 18 months after clinical discharge and for all health-, personal, and work-related factors. Second, Kaplan–Meier survival plots were used to estimate unadjusted median times and interquartile ranges with 95% confidence intervals (95% CI) from the date of discharge until first RTW and full RTW in calendar days for the total sample and each treatment setting. Log-rank tests were used to detect significant differences between the two survivor functions of each categorical variable. Third, parametric survival analysis [50–52] was applied to estimate coefficients with 95% CI for time to first RTW and full RTW after clinical discharge. For participants who did not return to work within the study period (*n* = 9), data were right-censored. The baseline survivor function of the empty (null) model was compared with the various given parametric distribution types in Stata [53]. The null model with the best fit (lowest Akaike information criterion [AIC] and Bayesian information criterion [BIC]) was chosen [53]. Based on the AIC and BIC for the null model, a ggamma survival function was preferred over the other calculated survival functions (see Supplementary Material 1 for the results of all null models) and used in all survival analyses.

Univariable survival analysis was conducted for time to first RTW and full RTW after clinical discharge for all health-related, personal, and work-related factors separately. Subsequently, a multivariable analysis was conducted for both outcomes, based on the understanding of the literature and the qualitative study findings [35]. To avoid or at least contain overfitting of the multivariable model effectively, a maximum set of nine variables was carefully selected. The following health-, personal, and work-related factors were included in the multivariable models: previous sickness absence and treatment duration, general health (*good*), depressive symptoms, RTW-SE (*high*), RTW expectation (*positive*), quality of leadership, social support, and individual RTW support needed (*yes*). All multivariable models were adjusted for age, sex, socioeconomic status and treatment setting. Negative coefficients reflected a shorter time to first RTW or full RTW and positive coefficients a longer time to first RTW or full RTW. To better reflect the differences in the sample characteristics, all survival analyses were repeated post hoc for both treatment settings separately. Stata 16.0 was used for all analyses (StataCorp. 2019. *Stata Statistical Software: Release 16*. College Station, TX: StataCorp LLC).

## Results

### Sample Characteristics

The total study sample consisted of 269 participants, *n* = 157 from inpatient psychiatric treatment and *n* = 112 from inpatient medical rehabilitation treatment (Table 1). Rehabilitation participants were approximately three years older and reported, on average, better overall health, higher work ability and RTW-SE scores, more positive RTW expectations and less work accommodation needs than psychiatric participants. A total of 66.1% (*n* = 74) of the rehabilitation participants, compared to 33.1% (*n* = 52) of the psychiatric participants, were not on sickness absence directly before the start of treatment. The average treatment duration of medical rehabilitation was two weeks shorter than psychiatric treatment. Whereas medical rehabilitation was organised as inpatient treatment for almost all participants (*n* = 110; 98.2%), the majority of psychiatric participants received day hospital treatment (*n* = 113; 72.0%).

**Table 1:** Sample characteristics and median time to first and full RTW after clinical discharge in calendar days for the total sample and each treatment setting

	Total sample (*N* = 269)			Psychiatric setting (*n* = 157)			Rehabilitation setting (*n* = 112)		
	N (%) or mean (SD)	Median time^a^ to first RTW	Median time^a^ to full RTW	N (%) or mean (SD)	Median time^a^ to first RTW	Median time^a^ to full RTW	N (%) or mean (SD)	Median time^a^ to first RTW	Median time^a^ to full RTW
Treatment setting									
Psychiatric	157 (58.4)	17***	73***						
Rehabilitation	112 (41.6)	6	6						
Gradual RTW during re-entry									
No	122 (45.4)	6***	6***	39 (24.8)	13	13	83 (74.1)	4**	4***
Yes	147 (54.6)	17	74	118 (75.2)	19	80	29 (25.9)	11	55
*Sociodemographics*									
Age [years]	48.2 (8.3)			46.9 (8.6)			50.2 (7.7)		
Sex									
Female	126 (46.8)	12	34	71 (45.2)	19	74	55 (49.1)	6	6*
Male	143 (53.2)	17	52	86 (54.8)	17	73	57 (50.9)	6	6
Socioeconomic status [3–21]	14.3 (3.4)			14.8 (3.5)			13.7 (3.3)		
*Health-related factors*									
Previous sickness absence duration [weeks]	5.6 (7.7)			6.5 (7.5)			4.3 (7.8)		
Treatment duration [weeks]	6.6 (1.8)			7.6 (1.6)			5.2 (0.8)		
Previous sickness absence and treatment duration [weeks]	12.2 (8.1)			14.1 (7.7)			9.6 (7.8)		
Self-rated health									
Good	206 (76.6)	11***	34***	114 (72.6)	17***	59***	92 (82.1)	6*	6**
Poor	63 (23.4)	31	80	43 (27.4)	39	115	20 (17.9)	6	13
Depressive symptoms [0–24]	7.7 (4.6)			8.1 (4.6)			7.0 (4.6)		
First medical diagnosis									
Depression or anxiety disorder	228 (84.8)	17***	52***	157 (100)	17	73	71 (63.4)	6	6
Adjustment disorder	41 (15.2)	6	6	0	–	–	41 (36.6)	6	6
Medical treatment									
No	164 (61.0)	11***	34*	86 (54.8)	17**	66*	78 (69.6)	6	6
Yes	105 (39.0)	17	55	71 (45.2)	24	80	34 (30.4)	6	7
Work-relatedness of disorder [1–5]	3.8 (1.1)			3.9 (1.0)			3.6 (1.1)		
*Personal factors*									
RTW expectation									
Positive	226 (84.0)	11***	32***	123 (78.3)	17***	59***	103 (92.0)	5***	6***
Negative	43 (16.0)	118	192	34 (21.7)	124	192	9 (8.0)	48	137
RTW-SE [1–6]	4.2 (1.1)			4.0 (1.1)			4.6 (1.0)		
High	120 (44.6)	6***	13***	51 (32.5)	15***	38***	69 (61.6)	6	6**
Low	149 (55.4)	17	73	106 (67.5)	24	80	43 (38.4)	6	12
Work ability [0–10]	5.3 (2.2)			4.7 (2.1)			6.2 (2.0)		
*Work-related factors*									
Company size									
Small	83 (30.9)	6*	34*	41 (26.1)	17	59	42 (37.5)	5	6
Large	186 (69.1)	17	52	116 (73.9)	17	73	70 (62.5)	6	6
Enterprise sector									
Private	187 (69.5)	17	47	116 (73.9)	21*	73	71 (63.4)	6	6
Public	82 (30.5)	11	32	41 (26.1)	17	73	41 (36.6)	6	7
Shiftwork									
No	203 (75.5)	17	52	130 (82.8)	17	73	73 (65.2)	6	6
Yes	66 (24.5)	7	27	27 (17.2)	17	73	39 (34.8)	6	6
Civil servant									
No	251 (93.3)	13	45*	146 (93.0)	17	70*	105 (93.8)	6	6
Yes	18 (6.7)	20	90	11 (7.0)	27	206	7 (6.2)	6	34
Quantitative demands [0–100]	65.2 (16.9)			67.4 (17.2)			62.2 (16.0)		
Work-privacy conflict [0–100]	61.9 (24.7)			64.1 (25.3)			58.7 (23.6)		
Influence at work [0–100]	38.5 (18.0)			38.7 (17.7)			38.2 (18.6)		
Degrees of freedom at work [0–100]	51.9 (20.9)			53.1 (20.4)			50.3 (21.6)		
Meaning of work [0–100]	72.4 (18.3)			69.7 (17.5)			76.3 (18.9)		
Quality of leadership [0–100]	46.0 (22.4)			44.8 (22.4)			47.6 (22.4)		
Social support [0–100]	53.8 (19.2)			52.5 (19.1)			55.7 (19.3)		
Sense of community [0–100]	66.6 (19.7)			66.9 (19.4)			66.2 (20.2)		
Mobbing [1–5]	2.3 (1.1)			2.2 (1.1)			2.3 (1.0)		
*Work accommodation needs*									
Gradual RTW needed									
No	140 (52.0)	6***	6***	55 (35.0)	17	31*	85 (75.9)	6	6***
Yes	129 (48.0)	18	73	102 (65.0)	20	80	27 (24.1)	6	54
Workload reduction needed									
No	187 (69.5)	12	38	106 (67.5)	17	67	81 (72.3)	5	6
Yes	82 (30.5)	17	54	51 (32.5)	24	73	31 (27.7)	6	12
Individual RTW support needed									
No	233 (86.6)	11**	34***	128 (81.5)	17	70	105 (93.8)	6	6*
Yes	36 (13.4)	31	89	29 (18.5)	31	94	7 (6.2)	32	89
Change of workplace needed									
No	243 (90.3)	12*	45*	140 (89.2)	17	67	103 (92.0)	5*	6
Yes	26 (9.7)	32	105	17 (10.8)	76	118	9 (8.0)	17	34

^a^Only for categorical variables; significant log-rank tests for equality of survivor functions ***p < 0.001, **p < 0.01, *p < 0.05

Regarding ‘work orientation and RTW preparation during treatment’, 91.1% (*n* = 143) of the psychiatric participants and 83.0% (*n* = 93) of the rehabilitation participants reported that their professional activities were discussed with them during their clinical stay. Sixty-eight per cent (*n* = 106) of the psychiatric participants and 60.7% (*n* = 68) of the rehabilitation participants reported that they had talked about social conflicts in the workplace during their clinical stay. Eighty-four per cent (*n* = 132) of the psychiatric participants and 55.4% (*n* = 62) of the rehabilitation participants reported that they had talked about potential problems with returning to work during their clinical stay. Seventy-five per cent of the psychiatric participants (*n* = 118) and 29.5% (*n* = 33) of the rehabilitation participants reported that their RTW process was prepared in the clinic with work accommodations like gradual RTW.

### Time to First and Full RTW

During the 18 months follow-up period after clinical discharge, *n* = 260 participants (96.7%) reported a first RTW. Nearly all of them (*n* = 252, 93.7%) achieved a full RTW. For the total sample, the median time to first RTW was 13 days [95% *CI* 11; 17] and 45 days [95% *CI* 34; 53] to full RTW. Among psychiatric participants, the median time to first RTW was 17 days [95% *CI* 17; 24] and 73 days [95% *CI* 59; 80] to full RTW. Among rehabilitation participants, the median time to both first RTW [95% *CI* 5; 6] and full RTW [95% *CI* 5; 11] was 6 days.

**Fig. 2:**
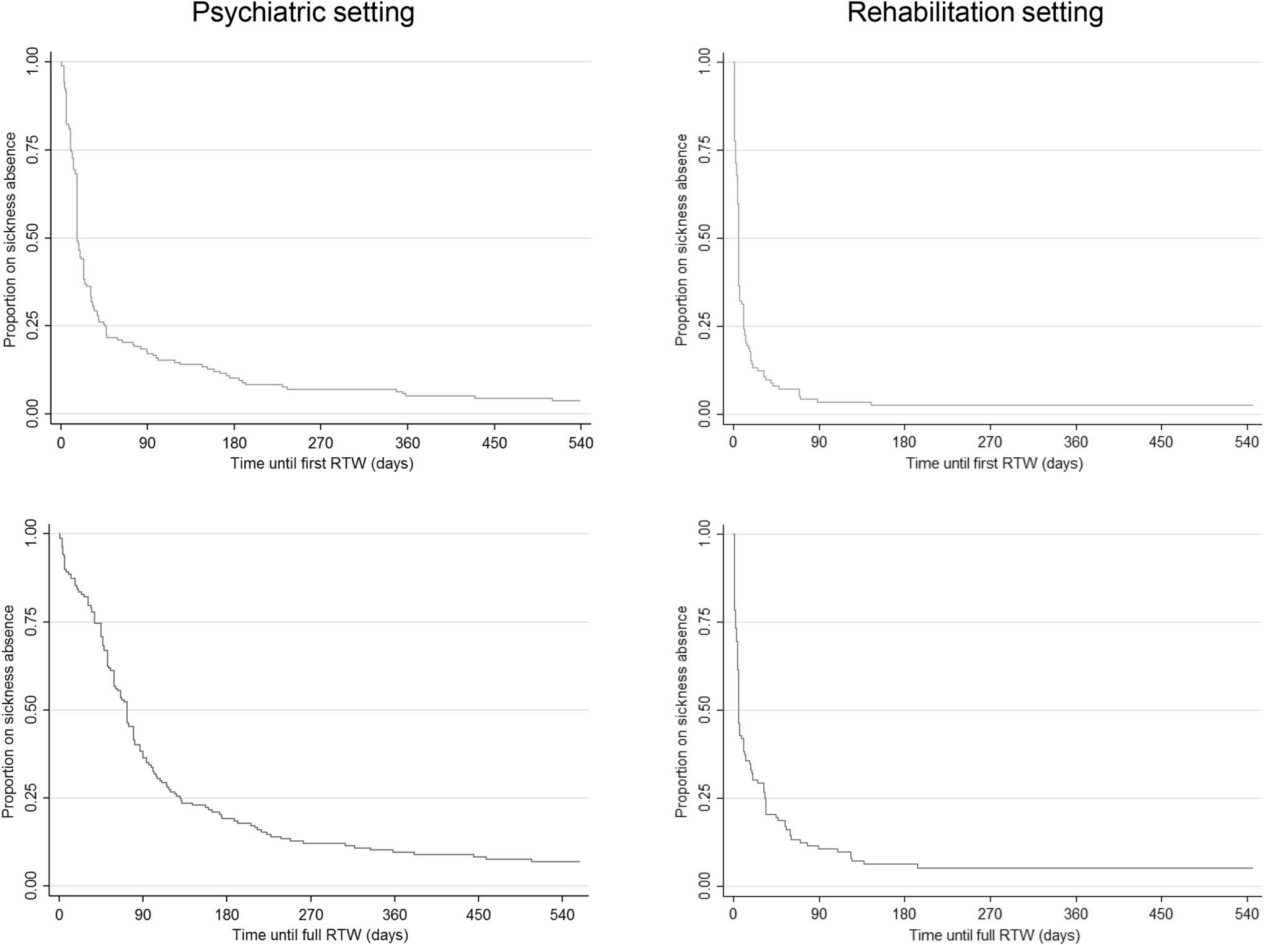
Time to first and full RTW for each treatment setting after clinical discharge in calendar days

In Fig. 2, the proportions of participants on sickness absence and the time until first RTW and full RTW are displayed for each treatment setting. A total of *n* = 147 participants (54.6%) started their re-entry (see Fig. 1) with a gradual RTW. The majority (*n* = 118; 80.3%) used a gradual RTW after psychiatric treatment. After medical rehabilitation treatment, *n* = 29 (19.7%) participants used a gradual RTW.

### Health-, Personal, and Work-Related Factors Associated with Time to First and Full RTW

Table 1 shows that the longest estimated median time until first RTW was found for participants with a negative RTW expectation in both treatment settings. The longest estimated median times until full RTW were found for participants who reported being a civil servant in the psychiatric setting and those with a negative RTW expectation in the rehabilitation setting. In the psychiatric setting, estimated median times to first and full RTW were considerably longer for those with poor self-rated health and low RTW-SE compared to those with a positive health perception and high RTW-SE. The results of the univariable parametric survival analysis for the total sample, and each treatment setting are shown in Supplementary Tables 1 and 2.

Table 2 illustrates that a longer duration of previous sickness absence and treatment, more depressive symptoms, and a negative RTW expectation at baseline were independently associated with a longer duration until first RTW. Additionally, low RTW-SE, worse quality of leadership (before treatment), and needed individual RTW support were independently associated with a longer duration until full RTW. Depressive symptoms were not associated with time to full RTW. In both settings, previous sickness absence and treatment duration were included, together with the RTW expectation until first RTW and needed individual RTW support until full RTW. In the psychiatric setting, good self-rated health and less depressive symptoms were also associated with a shorter time to first RTW. Moreover, in the psychiatric setting, high RTW-SE was associated with a shorter time to first and full RTW.

**Table 2:** Estimation results of the multivariable parametric survival analysis for the total sample and each treatment setting

	Total sample^a^			Psychiatric setting^b^			Rehabilitation setting^b,c^		
	Coef.	95% CI	P value	Coef.	95% CI	P value	Coef.	95% CI	P value
*Model ‘time to first RTW’*									
Previous sickness absence and treatment duration	0.05	0.04;0.07	0.000	0.05	0.03;0.07	0.000	0.07	0.03;0.10	0.000
Self-rated health (*good*)	− 0.36	− 0.73;0.01	0.056	− 0.52	− 0.90;− 0.14	0.007	− 0.39	− 1.11;0.34	0.294
Depressive symptoms	0.04	0.00;0.01	0.028	0.06	0.02;0.10	0.003	0.02	− 0.03;0.08	0.372
RTW-SE (*high*)	− 0.23	− 0.52;0.07	0.135	− 0.49	− .085;− 0.13	0.008	0.07	− 0.48;0.08	0.794
RTW expectation (*positive*)	− 1.00	− 1.40;− 0.60	0.000	− 0.99	− 1.39;− 0.59	0.000	− 1.51	− 2.50;− 0.52	0.003
Quality of leadership	− 0.00	− 0.01;0.00	0.438	− 0.01	− 0.01;0.00	0.269	− 0.00	− 0.02;0.01	0.867
Social support	0.00	− 0.01;0.01	0.903	0.00	− 0.01;0.01	0.836	0.00	− 0.02;0.02	0.869
Individual RTW support needed (*yes*)	0.29	− 0.10;0.67	0.144	0.35	− 0.04;0.73	0.078	0.35	− 0.65;1.34	0.496
Constant	2.43	1.05;3.81	0.001	2.48	0.61;4.35	0.009	1.87	− 0.74;4.47	0.160
Sigma (ln)	0.04	− 0.06;0.14	0.444	− 0.08	− 0.22;0.07	0.322	0.15	0.02;0.29	0.027
Number of observations	263			153			110		
AIC	813.58			453.94			371.67		
BIC	867.17			496.37			406.78		
*Model ‘time to full RTW’*									
Previous sickness absence and treatment duration	0.08	0.06;0.10	0.000	0.04	0.02;0.07	0.001	0.12	0.09;0.16	0.000
Self-rated health (*good*)	− 0.22	− 0.64;0.20	0.299	− 0.27	− 0.69;0.15	0.214	− 0.53	− 1.32;0.26	0.188
Depressive symptoms	0.01	− 0.03;0.05	0.582	0.01	− 0.04;0.05	0.706	− 0.01	− 0.07;0.05	0.739
RTW-SE (*high*)	− 0.59	− 0.93;− 0.25	0.001	− 0.79	− 1.17;− 0.41	0.000	− 0.28	− 0.89;0.32	0.362
RTW expectation (*positive*)	− 1.14	− 1.60;− 0.67	0.000	− 1.06	− 1.53;− 0.059	0.000	− 1.07	− 2.16;0.03	0.056
Quality of leadership	− 0.01	− 0.02;− 0.00	0.048	− 0.01	− 0.02;0.00	0.288	− 0.00	− 0.02;0.01	0.650
Social support	0.00	− 0.01;0.01	0.701	− 0.01	− 0.02;0.01	0.373	0.00	− 0.02;0.02	0.815
Individual RTW support needed (*yes*)	0.66	0.22;1.10	0.004	0.47	0.03;0.92	0.038	1.37	0.26;2.47	0.015
Constant	4.07	2.55;5.60	0.000	5.31	3.44;7.18	0.000	2.23	− 0.62;5.09	0.125
Sigma (ln)	0.18	0.10;0.27	0.000	0.03	− 0.11;0.15	0.732	0.24	0.10;0.38	0.001
Number of observations	263			153			110		
AIC	855.97			465.18			386.76		
BIC	909.55			507.61			421.87		

*Coef.* coefficient, *CI* confidence interval

^a^Adjusted for age, sex, socioeconomic status and treatment setting

^b^Adjusted for age, sex and socioeconomic status

^c^Here, the lognormal regression model was applied, because Stata could not compute an improvement when using the ggamma regression model

Table 3 provides a summary of the results for the total sample and each treatment setting.

**Table 3:** Summary of results of factors associated with a shorter or longer duration until first and full RTW

	Time to first RTW			Time to full RTW		
	Total sample	Psychiatric setting	Rehabilitation setting	Total sample	Psychiatric setting	Rehabilitation setting
*Univariable analysis*^a^						
Sociodemographics						
Age						
Sex (*male*)						
Socioeconomic status	o					
Health-related factors						
Previous sickness absence and treatment duration	o	o	o	o	o	o
Self-rated health (*good*)	•	•		•	•	•
Depressive symptoms	o	o		o	o	
First medical diagnosis (*adjustment*)	•			•		
Medical treatment (*yes*)	o			o	o	
Work-relatedness of disorder	o			o	o	o
Personal factors						
RTW expectation (*positive*)	•	•	•	•	•	•
RTW-SE (*high*)	•	•		•	•	•
Work ability	•	•		•	•	•
Work-related factors						
Company size (*large*)	o			o		
Enterprise sector (*private*)						
Shiftwork (*yes*)						
Civil servant (*yes*)				o	o	
Quantitative demands	o			o		
Work-privacy conflict	o			o	o	o
Influence at work						
Degrees of freedom at work						
Meaning of work	•			•	•	
Quality of leadership	•			•		
Social support	•	•		•	•	
Sense of community		•		•	•	
Mobbing	o	o			o	
Work accommodation needs						
Gradual RTW needed (*yes*)	o	o	o	o	o	o
Workload reduction needed (*yes*)	o		o			
Individual RTW support needed (*yes*)	o			o	o	o
Change of workplace needed (*yes*)	o		o	o		o
*Multivariable analysis*^a^						
Previous sickness absence and treatment duration	o	o	o	o	o	o
Self-rated health (*good*)		•				
Depressive symptoms	o	o				
RTW-SE (*high*)		•		•	•	
RTW expectation (*positive*)	•	•	•	•	•	
Quality of leadership				•		
Social support						
Individual RTW support needed (*yes*)				o	o	o

• = shorter duration until first/full RTW; o = longer duration until first/full RTW

^a^All categorical variables are displayed with the category referred to in brackets

## Discussion

This prospective cohort study among German employees investigated the time to first and full RTW and the associated factors after receiving inpatient treatment for CMDs. To the best of our knowledge, it is the first RTW study at the intersection between the mental healthcare system and workplace from the employees’ perspective in Germany. Almost all employees from both treatment settings, psychiatric treatment and medical rehabilitation, reported a first RTW and full RTW within the 18 month study period. While only health-related and personal factors were independently associated with time to first RTW, leadership quality and needed individual RTW support were associated with time to full RTW in the multivariable model.

The majority of employees receiving medical rehabilitation treatment returned to work very quickly, that is almost immediately after treatment. The first 50% of the rehabilitation participants needed only 1 week until achieving a first and full RTW and therefore had no re-entry phase. Next to the reported better health situation at baseline, this result may be explained by some specific characteristics of the rehabilitation system. Medical rehabilitation in Germany is provided mostly as inpatient treatment far from home and the workplace [34]. Access to medical rehabilitation has to be granted by the German Pension Insurance, and use of gradual RTW within four weeks after rehabilitation has to be financed by the German Pension Insurance. Recently, a prospective cohort study of the German Pension Insurance found that gradual RTW after medical rehabilitation is only effective with certain indication criteria, such as prior sickness absence of more than 12 weeks [54]. When not being on sickness absence directly before starting treatment (as two-thirds of the rehabilitation setting were in the present study), usually one will start and leave medical rehabilitation treatment with the status ‘able to work’ [55]. This status may lead to the assumption that there is no need for more RTW preparation during treatment (e.g., work accommodations for RTW) or time ‘off work’ afterwards.

Although the median time to full RTW was nine weeks longer when receiving psychiatric treatment compared to medical rehabilitation, the employees’ full RTW rates were just as successful. The majority of employees receiving psychiatric treatment had a day hospital stay; they reported more work orientation and RTW preparation during treatment, and many of them returned to work gradually. Two recent descriptive surveys of inpatient psychiatric care in Germany showed that patients’ employment rates were relatively low (21–34%), with a high need for RTW support [56, 57]. In one study, 71% of the employed patients returned to work within three months after treatment; 45% spoke about a gradual RTW opportunity during treatment, and 40% returned to work gradually [56]. In comparison, in the present study sample, RTW rates as well as the work orientation and RTW preparation during treatment seemed to be very high.

While acknowledging the different aims of the two health service providers, it was surprising to observe how much the times until RTW varied across the treatment settings. Hence, post hoc subgroup analyses were conducted with the two treatment settings.

The findings from this RTW study in the German social security system with two mental health service providers are in line with previous research from other countries [15, 16, 22]. Leadership quality and needed individual RTW support were associated with time to full RTW. Though expected based on the qualitative study findings [35], social support was not associated with either time to first or full RTW. Work accommodation needs were associated with a longer time to first or full RTW. When work accommodation needs are required, such as individual RTW support, an underlying complex problem situation is often present. In such cases, a more specific RTW preparation may be needed during treatment to enable a timelier RTW. In the rehabilitation setting, only work-privacy conflict was associated with a longer time to full RTW. Based on the qualitative findings [35], it may be speculated that rehabilitation participants tend to experience their working conditions as unchangeable, perhaps because the treatment is conducted far away from the workplace [34]. It may also explain why rehabilitation participants returned to work more often directly after treatment and less often reflecting on their working situation in the clinic, e.g. when preparing their RTW.

By addressing the two RTW outcomes ‘time to first RTW’ and ‘time to full RTW’, based on the model of Young et al. [14], the corresponding RTW phases (off work and re-entry) could be examined more closely. Whereas previous research focused primarily on time to full RTW [22], the segmentation into first and full RTW was essential to gain a deeper understanding of each setting, especially concerning the ‘off work’ and ‘re-entry’ phases, and in the end of the developmental nature of the RTW process and its progress [14].

Hence, for future research, a phase-specific approach to the entire RTW process is recommended. Additionally, given the multifactorial and complex nature of RTW, work-related factors and work outcomes should be consistently incorporated when investigating clinical study populations with CMDs over time in Germany. As the present study focused on the phases towards RTW, further research is needed on the long-term sustainability of RTW, also regarding possible long-term effects of gradual RTW and other work accommodations during RTW. Because social support was regarded an essential factor during the RTW process in the qualitative findings but was not associated with time to first and full RTW in the present study, it should be addressed in future trajectory analyses.

The present study identified some aspects where a stronger RTW focus in clinical practice could help support a timely RTW for employees receiving inpatient treatment for CMDs in the future. In particular, employees with a negative RTW expectation should be given more attention and support in their RTW process. As Nieuwenhuijsen et al. [43] stated, it can be helpful to systematically assess the RTW expectation at the beginning of the treatment or sickness absence to provide more comprehensive and tailored support towards RTW. The easy assessment of the RTW expectation during inpatient treatment could therefore be used to detect employees with CMDs at risk for a late RTW at an early stage and to provide continuous support in their RTW process. In the present study, high RTW-SE was associated with a considerably shorter time to full RTW. As recommended previously [43, 45], the RTW-SE questionnaire may be used to identify employees with CMDs at risk of a late RTW.

For employers and key RTW stakeholders at the workplace, the results highlight the importance of providing a good leadership quality in the RTW process, which has also been determined as a critical preventive resource for restoring mental health [20]. The results may also help to raise greater awareness at the workplace concerning the provision of tailored individual RTW support to enable a timelier RTW, especially in cases of longer sickness absence. In Germany, a specific operational integration management programme (*Betriebliches Eingliederungsmananagement ‘BEM’*) is regulated by law [27]. The programme offers the opportunity to address at an early stage the needs of the returning employees, who were on sickness absence of more than 6 weeks during the last 12 months. However, this programme is often used too late and not yet consistently implemented [58, 59].

For the medical rehabilitation setting, where the goal is to restore work and daily life capacity and to prevent permanent work disability, the results suggest that the predominantly inpatient structures away from home may offer room for improvement. With more flexible day hospital options' ‘at home' and more work orientation during treatment, the medical rehabilitation setting may contribute to a stronger connection with the workplaces in the future [34].

To develop targeted interventions for employees at risk of long-term sickness absence and permanent work disability, modifiable aspects such as RTW expectation, individual RTW support as needed work accommodation, or RTW-SE could be used.

## Strengths and Limitations

Strengths of this study are the prospective design with 18 months follow-up, the investigation of several areas of RTW factors simultaneously, and the focus on work accommodations needs for RTW from the perspective of the returning employees. Nevertheless, several limitations must be mentioned. First, all data, including the RTW outcomes, were self-reported, because no national-level register data on sickness absence are available in Germany. Hence, common method variance cannot be excluded. The medical first and second diagnoses, however, were reported by clinicians. Second, the results of the post hoc analyses should be treated with caution because of a lack of statistical power. Third, possible recall bias may have occurred because of the retrospective measurement of work factors and outcomes. Fourth, there may also have been selection bias regarding the comparability of the treatment settings and a healthy entrance effect due to the narrow inclusion criteria. Fifth, because treatment was not the focus of the study, information on the types of treatment was not assessed.

## Conclusions

The time and paths back to work after inpatient treatment due to CMDs varied across treatment settings. More attention to work accommodation needs for RTW in clinical practice, and the workplace may help to enable a timely RTW. While clinical practice might help mainly in preparing RTW at an early stage, a stronger connection and collaboration with key RTW stakeholders in the workplace may allow and promote the realisation of a coordinated and timely RTW.

## Supplementary Information

Below is the link to the electronic supplementary material.

Supplementary file1 (PDF 96 kb)

Supplementary file2 (PDF 145 kb)

## Data Availability

The datasets analysed in the present study are available from the corresponding author on reasonable request after official permission by the privacy officer at the Federal Institute for Occupational Safety and Health.

## Abbreviations

AIC: Akaike information criterion
BIC: Bayesian information criterion
CMD: Common mental disorder
RTW: Return to work
RTW-SE: Return to work self-efficacy
