# Supplementary material for: Employees Receiving Inpatient Treatment for Common Mental Disorders in Germany: Factors Associated with Time to First and Full Return to Work
Source: J Occup Rehabil. 2021 May 29;32(1):114–27. doi: 10.1007/s10926-021-09985-4 (PMC8858919; doi:10.1007/s10926-021-09985-4)
Supplement: Supplementary file 1 — Supplementary file1 (PDF 96 kb) [file 10926_2021_9985_MOESM1_ESM.pdf]

Title: Employees receiving inpatient treatment for common mental disorders in Germany: Factors associated with time to first and full return to work; Journal of Occupational Rehabilitation;

Authors: Alexandra Sikora, Gundolf Schneider, Uta Wegewitz, Ute Bültmann;

Corresponding author: Alexandra Sikora, Federal Institute for Occupational Safety and Health (BAuA), Sikora.Alexandra@baua.bund.de

Supplementary Material 1. Fitting null models.

1.1 Time to first RTW: Estimation results for several survival curves, no covariates

Akaike's information criterion and Bayesian information criterion

Model	N	ll(null)	ll(model)	df	AIC	BIC
exponential	269	-640.7057	-640.7057	1	1283.411	1287.006
weibull	269	-543.0385	-543.0385	2	1090.077	1097.266
gompertz	269	.	-513.4695	2	1030.939	1038.128
lognormal	269	.	-502.4559	2	1008.912	1016.101
loglogistic	269	.	-499.183	2	1002.366	1009.555
ggamma	269	.	-494.4236	3	994.8472	1005.631

Note: BIC uses N = number of observations. See **[R] BIC note**.

Likelihood-ratio tests found statistically significant differences between the loglogistic, lognormal and the ggamma function ($p < .01$; $p < .001$). The ggamma model best described the time to first RTW.

1.2 Time to full RTW: Estimation results for several survival curves, no covariates

Akaike's information criterion and Bayesian information criterion

Model	N	ll(null)	ll(model)	df	AIC	BIC
exponential	269	-616.739	-616.739	1	1235.478	1239.073
weibull	269	-549.6345	-549.6345	2	1103.269	1110.458
gompertz	269	.	-552.9167	2	1109.833	1117.023
lognormal	269	.	-540.6879	2	1085.376	1092.565
loglogistic	269	.	-544.7304	2	1093.461	1100.65
ggamma	269	.	-540.5368	3	1087.074	1097.858

Note: BIC uses N = number of observations. See **[R] BIC note**.

Based on the AIC and BIC for the empty model, a lognormal survival function would be most preferable, but a likelihood-ratio test found no significant differences between the lognormal and the ggamma function ($p = 0.58$). It was, therefore, continued with the ggamma survival function.
