# Supplementary material for: Employees Receiving Inpatient Treatment for Common Mental Disorders in Germany: Factors Associated with Time to First and Full Return to Work
Source: J Occup Rehabil. 2021 May 29;32(1):114–27. doi: 10.1007/s10926-021-09985-4 (PMC8858919; doi:10.1007/s10926-021-09985-4)
Supplement: Supplementary file 2 — Supplementary file2 (PDF 145 kb) [file 10926_2021_9985_MOESM2_ESM.pdf]

Title: Employees receiving inpatient treatment for common mental disorders in Germany: Factors associated with time to first and full return to work; Journal of Occupational Rehabilitation;

Authors: Alexandra Sikora, Gundolf Schneider, Uta Wegewitz, Ute Bültmann;

Corresponding author: Alexandra Sikora, Federal Institute for Occupational Safety and Health (BAuA), Sikora.Alexandra@baua.bund.de

Supplementary Table 1. Estimation results of the univariable parametric survival analysis.

		Time to first RTW					Time to full RTW				
	n	Coef.	95% CI			P value	Coef.	95% CI			P value
Treatment setting (*rehabilitation*)	269	-1.46	-1.75	;	-1.16	0.000	-2.09	-2.48	;	-1.71	0.000
Sociodemographics											
Age	269	-0.01	-0.03	;	0.01	0.429	-0.02	-0.04	;	0.01	0.246
Sex (*male*)	269	0.15	-0.20	;	0.51	0.394	0.28	-0.17	;	0.74	0.223
Socioeconomic status	266	0.06	0.01	;	0.11	0.021	0.04	-0.03	;	0.11	0.261
Health-related factors											
Previous sickness absence and treatment duration	269	0.09	0.07	;	0.11	0.000	0.13	0.11	;	0.15	0.000
Self-rated health (*good*)	269	-1.07	-1.55	;	-0.59	0.000	-1.34	-1.85	;	-0.83	0.000
Depressive symptoms	269	0.09	0.05	;	0.13	0.000	0.09	0.04	;	0.14	0.000
First medical diagnosis (*adjustment*)	269	-1.15	-1.63	;	-0.68	0.000	-1.54	-2.14	;	-0.94	0.000
Medical treatment (*yes*)	269	0.50	0.14	;	0.87	0.007	0.64	0.18	;	1.10	0.007
Work-relatedness of disorder	268	0.25	0.09	;	0.41	0.002	0.41	0.19	;	0.62	0.000
Personal factors											
RTW expectation (*positive*)	269	-2.15	-2.61	;	-1.69	0.000	-2.37	-2.97	;	-1.77	0.000
RTW-SE (*high*)	269	-0.91	-1.26	;	-0.56	0.000	-1.52	-1.94	;	-1.11	0.000
Work ability	269	-0.38	-0.46	;	-0.30	0.000	-0.47	-0.55	;	-0.38	0.000
Work-related factors											
Company size (*large*)	269	0.39	0.01	;	0.77	0.046	0.52	0.04	;	1.01	0.035
Enterprise sector (*private*)	269	0.27	-0.11	;	0.65	0.166	0.23	-0.27	;	0.72	0.366
Shiftwork (*yes*)	269	-0.35	-0.76	;	0.05	0.088	-0.46	-0.99	;	0.07	0.088
Civil servant (*yes*)	269	0.54	-0.16	;	1.24	0.133	1.20	0.29	;	2.10	0.010
Quantitative demands	268	0.01	0.00	;	0.03	0.008	0.02	0.00	;	0.03	0.014
Work-privacy conflict	268	0.01	0.00	;	0.02	0.002	0.02	0.01	;	0.02	0.001
Influence at work	269	-0.00	-0.01	;	0.01	0.739	-0.01	-0.02	;	0.01	0.248
Degrees of freedom at work	269	-0.00	-0.01	;	0.01	0.495	-0.01	-0.02	;	0.00	0.140
Meaning of work	269	-0.01	-0.02	;	-0.00	0.026	-0.02	-0.03	;	-0.01	0.001
Quality of leadership	266	-0.01	-0.02	;	-0.00	0.039	-0.01	-0.02	;	-0.00	0.029
Social support	268	-0.01	-0.02	;	-0.00	0.004	-0.02	-0.03	;	-0.01	0.001
Sense of community	268	-0.01	-0.02	;	0.00	0.064	-0.02	-0.03	;	-0.00	0.005
Mobbing	268	0.18	0.01	;	0.36	0.038	0.21	-0.00	;	0.42	0.053
Work accommodation needs											
Gradual RTW needed (*yes*)	269	1.09	0.78	;	1.41	0.000	2.15	1.77	;	2.53	0.000
Workload reduction needed (*yes*)	269	0.44	0.06	;	0.82	0.025	0.44	-0.07	;	0.95	0.090
Individual RTW support needed (*yes*)	269	0.94	0.43	;	1.45	0.000	1.44	0.78	;	2.11	0.000
Change of workplace needed (*yes*)	269	1.05	0.46	;	1.63	0.000	0.98	0.21	;	1.75	0.012

Coef. Coefficient; *CI* Confidence Interval

Supplementary Table 2. Estimation results of the univariable parametric post hoc subgroup survival analysis.

	Psychiatric setting							Rehabilitation setting						
		Time to first RTW			Time to full RTW				Time to first RTW			Time to full RTW[1]		
	n	Coef.	95% CI	P value	Coef.	95% CI	P value	n	Coef.	95% CI	P value	Coef.	95% CI	P value
Sociodemographics														
Age	157	0.02	-0.00 ; 0.04	0.064	0.02	-0.00 ; 0.05	0.058	112	0.00	-0.03 ; 0.03	0.996	-0.03	-0.08 ; 0.01	0.146
Sex (*male*)	157	0.09	-0.31 ; 0.48	0.672	-0.12	-0.54 ; 0.30	0.575	112	0.08	-0.32 ; 0.49	0.696	0.59	-0.09 ; 1.27	0.091
Socioeconomic status	155	0.04	-0.02 ; 0.10	0.173	-0.00	-0.06 ; 0.06	0.998	111	0.02	-0.04 ; 0.08	0.434	-0.03	-0.13 ; 0.08	0.606
Health-related factors														
Previous sickness absence and treatment duration	157	0.06	0.04 ; 0.08	0.000	0.07	0.04 ; 0.10	0.000	112	0.08	0.06 ; 0.10	0.000	0.15	0.12 ; 0.19	0.000
Self-rated health (*good*)	157	-0.98	-1.43 ; -0.53	0.000	-0.95	-1.39 ; -0.50	0.000	112	-0.08	-0.65 ; 0.49	0.781	-1.09	-1.98 ; -0.20	0.016
Depressive symptoms	157	0.10	0.05 ; 0.14	0.000	0.08	0.03 ; 0.12	0.001	112	0.02	-0.03 ; 0.07	0.476	0.04	-0.03 ; 0.12	0.277
First medical diagnosis (*adjustment*)	157	-	- ; -	-	-	- ; -	-	112	-0.27	-0.69 ; 0.15	0.212	-0.24	-0.95 ; 0.47	0.511
Medical treatment (*yes*)	157	0.24	-0.17 ; 0.65	0.257	0.49	0.08 ; 0.90	0.019	112	0.15	-0.30 ; 0.61	0.517	0.12	-0.63 ; 0.87	0.748
Work-relatedness of disorder	157	0.11	-0.09 ; 0.30	0.285	0.27	0.05 ; 0.49	0.016	111	0.19	-0.01 ; 0.38	0.058	0.36	0.06 ; 0.65	0.019
Personal factors														
RTW expectation (*positive*)	157	-1.64	-2.13 ; -1.15	0.000	-1.42	-1.90 ; -0.93	0.000	112	-1.95	-2.74 ; -1.17	0.000	-3.17	-4.35 ; -1.99	0.000
RTW-SE (*high*)	157	-0.88	-1.26 ; -0.50	0.000	-1.02	-1.47 ; -0.58	0.000	112	-0.13	-0.56 ; 0.29	0.540	-0.96	-1.65 ; -0.27	0.006
Work ability	157	-0.34	-0.42 ; -0.26	0.000	-0.35	-0.45 ; -0.26	0.000	112	-0.08	-0.19 ; 0.03	0.158	-0.34	-0.50 ; -0.17	0.000
Work-related factors														
Company size (*large*)	157	0.12	-0.32 ; 0.57	0.592	0.15	-0.33 ; 0.62	0.543	112	0.13	-0.30 ; 0.56	0.546	0.37	-0.33 ; 1.08	0.301
Enterprise sector (*private*)	157	0.41	-0.03 ; 0.85	0.069	0.12	-0.35 ; 0.59	0.615	112	-0.04	-0.47 ; 0.38	0.844	-0.18	-0.90 ; 0.53	0.615
Shiftwork (*yes*)	157	-0.48	-1.03 ; 0.07	0.087	-0.01	-0.58 ; 0.55	0.961	112	0.13	-0.29 ; 0.55	0.553	0.12	-0.60 ; 0.84	0.744
Civil servant (*yes*)	157	0.52	-0.24 ; 1.28	0.180	1.08	0.26 ; 1.89	0.010	112	0.51	-0.30 ; 1.33	0.216	1.11	-0.31 ; 2.53	0.125
Quantitative demands	157	0.01	-0.00 ; 0.02	0.186	0.01	-0.00 ; 0.02	0.155	111	0.01	-0.01 ; 0.02	0.408	0.01	-0.01 ; 0.03	0.513
Work-privacy conflict	157	0.01	-0.00 ; 0.02	0.062	0.01	0.00 ; 0.02	0.015	111	0.01	-0.00 ; 0.02	0.154	0.02	0.00 ; 0.03	0.025
Influence at work	157	0.00	-0.01 ; 0.01	0.857	-0.00	-0.02 ; 0.01	0.447	112	-0.00	-0.01 ; 0.01	0.605	-0.01	-0.03 ; 0.01	0.221
Degrees of freedom at work	157	-0.01	-0.02 ; 0.05	0.271	-0.01	-0.02 ; -0.00	0.012	112	-0.01	-0.01 ; 0.00	0.284	-0.01	-0.03 ; 0.01	0.241
Meaning of work	157	-0.01	-0.02 ; -0.00	0.050	-0.01	-0.03 ; -0.00	0.022	112	0.00	-0.01 ; 0.01	0.833	-0.01	-0.03 ; 0.01	0.399
Quality of leadership	155	-0.01	-0.02 ; 0.00	0.078	-0.00	-0.02 ; 0.00	0.068	111	-0.01	-0.01 ; 0.00	0.154	-0.01	-0.02 ; 0.01	0.410
Social support	157	-0.01	-0.02 ; -0.00	0.020	-0.02	-0.03 ; -0.01	0.000	111	-0.01	-0.02 ; 0.00	0.089	-0.01	-0.03 ; 0.01	0.263

Sense of community	157	-0.01	-0.02 ; -0.00	0.011	-0.02	-0.03 ; -0.01	0.001	111	-0.00	-0.01 ; 0.01	0.426	-0.01	-0.03 ; 0.00	0.102
Mobbing	157	0.25	0.07 ; 0.43	0.006	0.20	0.01 ; 0.39	0.040	111	0.16	-0.06 ; 0.39	0.148	0.32	-0.04 ; 0.68	0.079
Work accommodation needs														
Gradual RTW needed (*yes*)	157	0.59	0.19 ; 0.98	0.003	1.25	0.76 ; 1.75	0.000	112	0.54	0.06 ; 1.03	0.029	1.88	1.16 ; 2.60	0.000
Workload reduction needed (*yes*)	157	0.08	-0.34 ; 0.50	0.703	0.15	-0.29 ; 0.60	0.504	112	0.57	0.12 ; 1.02	0.013	0.57	-0.19 ; 1.33	0.144
Individual RTW support needed (*yes*)	157	0.32	-0.21 ; 0.84	0.234	0.54	0.00 ; 1.07	0.049	112	0.64	-0.31 ; 1.60	0.185	2.66	1.31 ; 4.01	0.000
Change of workplace needed (*yes*)	157	0.47	-0.18 ; 1.12	0.159	0.41	-0.26 ; 1.08	0.229	112	1.32[1]	0.36 ; 2.27	0.007	1.72	0.49 ; 2.96	0.006

Coef. Coefficient; *CI* Confidence Interval; [1] Here, the lognormal regression model was applied, because Stata could not compute an improvement when using the ggamma regression model
